# Kai-Xin-San, a Chinese Herbal Decoction Containing Ginseng Radix et Rhizoma, Polygalae Radix, Acori Tatarinowii Rhizoma, and Poria, Stimulates the Expression and Secretion of Neurotrophic Factors in Cultured Astrocytes

**DOI:** 10.1155/2013/731385

**Published:** 2013-10-03

**Authors:** Kevin Yue Zhu, Sherry Li Xu, Roy Chi-Yan Choi, Artemis Lu Yan, Tina Ting-Xia Dong, Karl Wah-Keung Tsim

**Affiliations:** ^1^Division of Life Science, Center for Chinese Medicine and State Key Laboratory of Molecular Neuroscience, The Hong Kong University of Science and Technology, Clear Water Bay Road, Hong Kong; ^2^Jiangsu Key Laboratory for High Technology Research of TCM Formulae and Jiangsu Collaborative Innovation Center of Chinese Medicinal Resources Industrialization, Nanjing University of Chinese Medicine, Nanjing, China

## Abstract

Kai-xin-san (KXS), a Chinese herbal decoction prescribed by Sun Simiao in *Beiji Qianjin Yaofang* about 1400 years ago, contains Ginseng Radix et Rhizoma, Polygalae Radix, Acori Tatarinowii Rhizoma, and Poria. In China, KXS has been used to treat stress-related psychiatric diseases with the symptoms of depression and forgetfulness. Although animal study has supported the antidepression function of KXS, the mechanism in cellular level is still unknown. Here, a chemically standardized water extract of KXS was applied onto cultured astrocytes in exploring the action mechanisms of KXS treatment, which significantly stimulated the expression and secretion of neurotrophic factors, including NGF, BDNF, and GDNF, in a dose-dependent manner: the stimulation was both in mRNA and protein levels. In addition, the water extracts of four individual herbs did not significantly stimulate the expression of neurotrophic factors, which could explain the optimized effect of KXS in a herbal decoction. The KXS-induced expression of neurotrophic factors did not depend on signaling mediated by estrogen receptor or protein kinase. The results suggested that the antidepressant-like action of KXS might be mediated by an increase of expression of neurotrophic factors in astrocytes, which fully supported the clinical usage of this decoction.

## 1. Introduction

Due to the fast speed of our daily life, more and more people are suffering from a depressive episode featured with these symptoms: (i) mood disturbance: anhedonia (loss of interest and pleasure), persistent depression, feeling helpless, or experiencing excessive guilt; (ii) cognitive disturbance: loss of memory and difficulty in concentrating; (iii) behavior disturbance: difficult in sleeping, loss of appetite or overeating, agitation, and suicidal tendency. If these symptoms occur together and last for more than two weeks without significant improvement, the result of major depression disorder (i.e., depression) will be diagnosed [1]. Now, depression has become one of the common psychiatric disorders, having the incidence of 15% of the total population and perhaps higher for women at 25% [2].

The deficiency of neurotransmitters, for example, norepinepherine, dopamine, and serotonin in brain, has long been regarded as the major cause of depression. Thus, all of the antidepression drugs available on the market are targeted on the restoration of decreased levels of neurotransmitters in synaptic cleft or in depressive brain by inhibiting the reuptake and degradation of neurotransmitters. However, 30%–40% of patients failed to respond to an initial 4–6-week treatment with an antidepression drug. Based on these phenomena, neurotrophic factor theory has been proposed [3]. Neurotrophic factors, including nerve growth factor (NGF), brain derived neurotrophic factor (BDNF), glial derived neurotrophic factor (GDNF), neurotrophin 3 (NT3), neurotrophin 4/5 (NT4/5) secreted from astrocytes, or target tissues, play a major role in neuron survival, as well as the synapse formation [4, 5]. Low level of BDNF had been discovered clinically in hippocampus and prefrontal cortex of depressive patients [6, 7]. Postmortem analyses of brain tissues from depressive patients showed a reduction of BDNF in brain and serum [8, 9]. On the other hand, brain infusion of BDNF produced antidepressant-like action in animals [10], as well as for NGF [11]. Therefore, the newly developed antidepression drugs should be designed aiming at multitargets, instead of a single neurotransmitter target.

Traditional Chinese medicine (TCM) offers a possible therapy for the treatment of depression, and a herbal decoction named Kai-Xin-San (KXS) is the most popular one. The first description of KXS is recorded in *Beiji Qianjin Yaofang* (*Thousand Formulae for Emergency*) written by Sun Simiao in Tang Dynasty (i.e., 652 A.D.). This herbal formula composes of four herbs: Ginseng Radix et Rhizoma (root and rhizome of *Panax ginseng* C. A. Mey.), Polygalae Radix (root of *Polygala tenuifolia* Wild.), Acori Tatarinowii Rhizoma (rhizome of *Acorus tatarinowii* Schott), and Poria (sclerotium of *Poria cocos* (Schw.) Wolf).Interestingly, at least three KXS formulae having a variation of herb ratio were described in ancient books, and all of them are commonly used clinically. In our previous study, we have demonstrated that KXS relieved depression-like symptoms on a chronic mild stress (CMS) induced depressive rat model by increasing the amounts of neurotransmitters and neurotrophic factors in the brain [12]. Here, we aimed to explore the mechanism of KXS in regulating neurotrophic factors in cultured astrocytes. In addition, the roles of different formulations of KXS and individual herb in the expression of neurotrophic factors were elucidated. 

## 2. Materials and Methods

### 2.1. Tissue Culture

Astrocytes from postnatal SD rat at day 1 were isolated and cultured. The cortex was dissected in Hank's Balanced Salt Solution without Ca^2+^ and Mg^2+^ (Sigma-Aldrich, St. Louis, MO). After being trypsinized for 15 min, the cortex was washed with culture medium and triturated several times. The culture medium was minimum essential medium (MEM) supplemented with 10% horse serum, 100 U/mL penicillin, and 100 *μ*g/mL streptomycin. All culture reagents were purchased from Invitrogen Technologies (Carlsbad, CA). The cells were centrifuged at 250 ×g for 5 min. The cell pellet was suspended in the culture medium. The cells were seeded on plastic culture plates with the density of 2 × 10^4^ cell/cm^2^ and incubated at 37°C in 95% air, 5% CO_2_. The culture medium was changed twice a week. Each time, the culture was pipetted up and down gently to remove loosely attached oligodendrocytes, microglia, and neurons.

### 2.2. Immunocytofluorescent Staining

Cultured astrocytes were grown on glass cover slip and fixed with 4% paraformaldehyde (PFA) in PBS for 15 min, followed by 50 mM ammonium chloride (NH_4_Cl) treatment for 25 min. Cultures were permeablized by 0.1% Triton X-100 in PBS for 10 min and blocked by 5% BSA in PBS for 1 hour at room temperature. The primary antibodies were then applied onto the cells for 16 hours at 4°C, which were mouse antineurofilament 68 antibody (1 : 200, Sigma-Aldrich), mouse antiglial fibrillary acidic protein (GFAP)-Cy3 (1 : 500, Sigma-Aldrich), and anti-rabbit anti-oligo2 (1 : 200, Imgenex). Then, the culture was stained with Alexa Fluor@ 488 donkey anti-Mouse antibody (1 : 1000, Invitrogen) and DAPI (1 : 500, Sigma-Aldrich) for 1 hour at room temperature. After being washed with PBS for 4 × 15 min, the cells were dehydrated serially with ice-cold 50%, 75%, 95%, and 100% ethanol and mounted with fluorescence mounting medium. Samples were then examined by Zeiss confocal microscopy with Ex 488/Em 505–540 nm for green color, Ex 543/Em 560–615 nm for Cy3 red color.

### 2.3. KXS Decoction Preparation

KXS decoction was composed of the following dried raw materials: Ginseng Radix et Rhizome (root and rhizome of *P. ginseng*), Polygalae Radix (root of *P. tenuifolia*), Acori Tatarinowii Rhizoma (rhizome of *A. tatarinowii*), and Poria (sclerotium of *P. cocos*). The herbs were purchased from Qingping Market of Chinese herbs in Guangzhou China and were authenticated by one of the authors, Dr. Tina T. X. Dong, according to their morphological characteristics. The voucher specimens were deposited in Centre for Chinese Medicine at Hong Kong University of Science & Technology. According to different formulations of KXS including KXS-652, KXS-984, and DZW-652, the appropriate amounts of Ginseng Radix et Rhizome, Polygalae Radix, Acori Tatarinowii Rhizoma, and Poria were weighed separately to form a combined weight of 20 g. The herbal extraction was performed in 160 mL of boiling water for 2 hours, and the herbs were extracted twice. For the second extraction of KXS, the residue from the first extraction was filtered: the same extraction condition was applied on the filtered residue. Then, the extracts were combined, dried under vacuum, and stored at −80°C. This extraction condition was also applied to each individual herb as well as a combination of herbs under the same extraction method as described above. The herbal extract was chemically standardized as reported previously [13], and the representative fingerprinting chromatograms were developed.

### 2.4. Drug Treatment

During the treatment, cultured astrocytes were changed with medium for 3 hours in modified Eagle's medium supplemented with 0.5% horse serum, 100 U/mL penicillin, and 100 *μ*g/mL streptomycin, and then the cultures were treated with KXS extracts and/or other reagents for 48 hours. In analyzing the signaling pathway, the cells were pretreated with the protein kinase A inhibitor H89 (2 *μ*M) and the MEK1/2 inhibitor U0126 (20 *μ*M) and ICI 182, 780 (1 *μ*M) for 3 hours before the exposure to KXS extract, Bt_2_-cAMP (1 mM), TPA (10 nM), and 17*β*-Estradiol (10 nM).

### 2.5. Total RNA Extraction

Total RNA from brain tissue was isolated with RNAzol reagent according to the manufacturer's protocol. The brain tissues were added with RNAzol reagent (1.5 mL/g) and homogenized. The homogenate was centrifuged at 16,100 ×g for 5 min at 4°C. The supernatants were removed, added with diethylpyrocarbonate (DEPC) treated water (prepared by autoclaving water with DEPC in a 1000 : 1 ratio) and vortex vigorously for 15 sec, followed by centrifugation at 16,100 ×g for 10 min at 4°C. The aqueous layer was collected and added with half volume of 70% ethanol in DEPC treated water for RNA precipitation. The RNA pallet was collected by centrifugation at 16,100 ×g for 10 min at 4°C and washed with 70% ethanol in DEPC-treated water twice. After air dry, the RNA was resuspended in 200 *μ*L of DEPC treated water. The concentration of extracted RNA was calculated from UV absorbance at 260 nm. The quality of RNA was assessed by absorbance at 260 nm and 280 nm, with the ratio of 260/280 nm ranging from 1.90 to 2.10 being acceptable.

### 2.6. Real-Time Quantitative PCR

Isolated RNAs were reverse transcribed by Moloney Murine Leukemia Virus (MMLV) reverse transcriptase with oligo-d(T) primer in a 20 *μ*L reaction by using High Capacity cDNA Reverse Transcription Kit of Invitrogen Technologies. In details, three *μ*g of total RNA was mixed with 1 *μ*L of 0.5 *μ*g/mL oligo-d(T) primer, 1 *μ*L of 10 mM dNTP mix, and RNAase/DNAase free water in a 12 *μ*L reaction. The mixture was incubated in 65°C for 5 min. Two *μ*L of 0.1 M dithiothreitol (DTT), 1 *μ*L of 40 U/*μ*L RNase out, and 4 *μ*L of 5x first strand buffer (250 mM Tris-HCl, pH 8.3, 375 mM KCl, and 15 mM MgCl_2_) were added into the reaction mix and incubated at 37°C for 5 min. One *μ*L MMLV was added into the reaction and incubated at 37°C for 50 min. Then, the reaction was incubated at 70°C for 15 min. Quantification of the cDNA was determined by UV absorbance at 260 nm and 280 nm by NanoDrop. 

Real-time quantitative PCR for the target genes was performed on equal amounts of cDNA by using Roche SYBR Green Master mix with Rox reference dye, according to the manufacturer's instructions. The SYBR green signal was detected by Applied Biosystems 7500 Fast Real-Time PCR System. Transcript levels were quantified by using the ΔΔCt value method. Calculation was done by using the Ct value of Glyceraldehyde 3-phosphate dehydrogenase (GAPDH) to normalize the Ct value of target gene in each sample to obtain the ΔCt value, which was then used to compare among different samples. Real-time PCR products were analyzed by gel electrophoresis on a 1.5% agarose gel, and the specificity of amplification was confirmed by the melting curves.

### 2.7. ELISA Analysis

The amounts of NGF, GDNF (both from Abfrontier, Seoul, Korea), and BDNF (Millipore, Billerica, MA) in astrocyte conditioned medium were measured using commercially available ELISA kits according to the manufacturer's instructions. Briefly, conditioned medium was applied onto a 96-well plate precoated with anti-neurotrophic factor antibodies and incubated on at 37°C for 90 min. After discarding plate content, the biotinylated anti-neurotrophic antibody was added and incubated at 37°C for 60 min. After washing four times, avidin-biotin-peroxidase, or streptavidin-peroxidase, complex solution was added and incubated at 37°C for 90 min. Tetramethylbenzidine solution was added and incubated at 37°C for 30 min. The reaction was stopped with 1 M sulfuric acid and absorbance recorded at 450 nm immediately. The values of standards and samples were corrected by subtracting the absorbance of nonspecific blinding. All samples were measured in duplicate in the same assay to minimize interassay variation.

### 2.8. Protein and Statistical Analysis

The concentration of protein was determined following the instructions of Bradford's method with a kit from Bio-Rad Laboratories. Individual data was expressed as mean ± standard deviation (SD). Statistical tests were performed with *t*-test (version 13.0, SPSS). Statistically significant changes were classified as significant (*) where *P* < 0.05, more significant (**) where *P* < 0.01, and highly significant (***) where *P* < 0.001.

## 3. Results

### 3.1. KXS Stimulates the Expression of Neurotrophic Factors on Astrocytes

**Figure 1:**
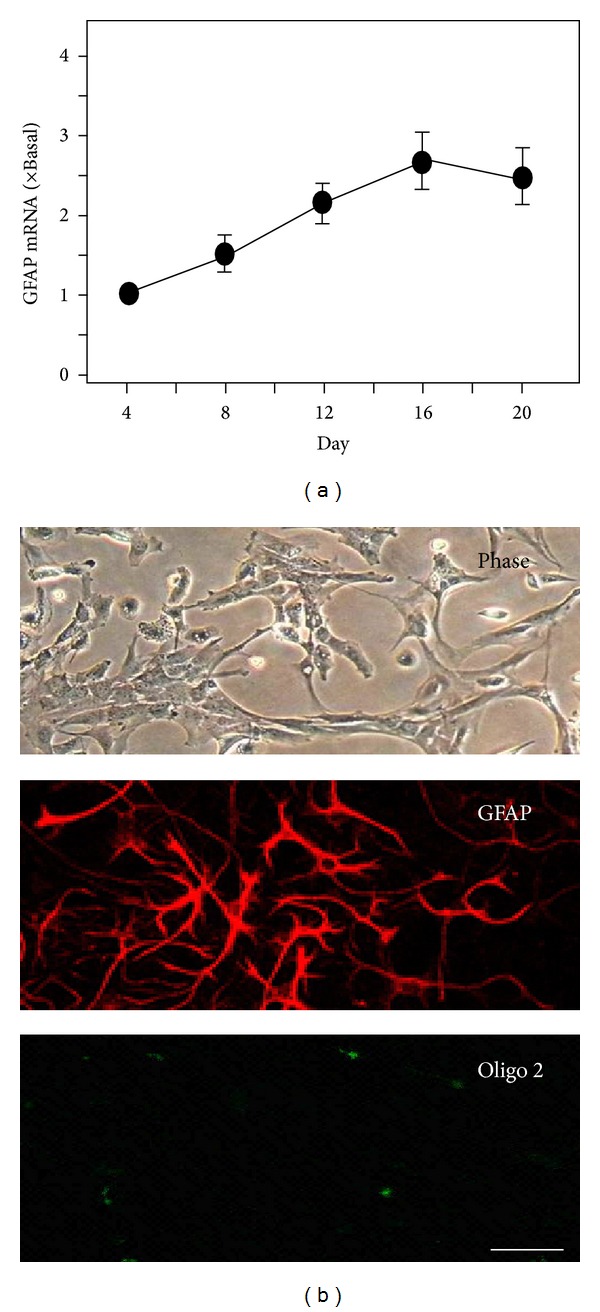
Cultures of rat astrocytes. (a) Cultured astrocytes were harvested on DIV4, 8, 12, 16, and 20, and the mRNA encoding GFAP was analyzed. Values are expressed as the percentage of increase to DIV 4 (set as 1) and in mean ± SEM, *n* = 3. (b) Cultured astrocytes were observed under the microscopy. Meanwhile, cultured astrocytes were stained by Cy3-anti-GFAP (shown in red) and anti-rabbit anti-oligo2 (shown in green) polyclonal antibodies observed under confocal microscopy in the presence of 0.1% Triton-X 100. Bar = 10 *μ*m. Representative figures are showed here, *n* = 3.

Cultured astrocytes reached confluence on the day in vitro (DIV) 12 having the mRNA level of glial fibrillary acidic protein (GFAP), a marker for astrocyte, reaching the peak on DIV 12 to 16 (Figure 1(a)). To test the culture purity, the astrocytes were seeded onto the glass cover slip. At DIV 12, the culture was fixed for immunofluorescent staining with GFAP and possible contamination of oligodendrocytes, which was viewed under the microscopy (Figure 1(b)). The result showed that the majority of cell population was astrocyte, and thus, this stage of astrocyte was used subsequently for all biochemical analyses.

Historically, three formulations of KXS have been described, denoted as KXS-652, KXS-984, and DZW-652 (Table 1), and all of them are commonly used today clinically. The notation was described according to the year of their publication. KXS-652 with a ratio of 1 : 1 : 25 : 50 of Ginseng Radix et Rhizoma : Polygalae Radix : Acori Tatarinowii Rhizoma : Poria. Meanwhile, a herbal formula named Ding-Zhi-Wan (DZW-652) was also described with the ratio of 3 : 2 : 2 : 3. In addition, KXS-984 was recorded in a herbal ratio of 1 : 1 : 1 : 2. The chemical standardizations of these herbal decoctions were done by HPLC fingerprints and quantitation of chemical ingredients (Supplementary Figure 1), as described fully in Zhu et al. [13]. By determining the amounts of marker chemicals from the herbs, a standardized KXS extract was recommended in containing minimal amounts of ginsenoside Rb_1_, ginsenoside Rd, ginsenoside Re, ginsenoside Rg_1,_ pachymic acid, 3, 6′-disinapoyl sucrose, *α*-asarone, and *β*-asarone (see Table 5 of [13] for detail). The chemical properties of these herbal extracts were prerequisite for all biochemical analyses.

**Figure 2:**
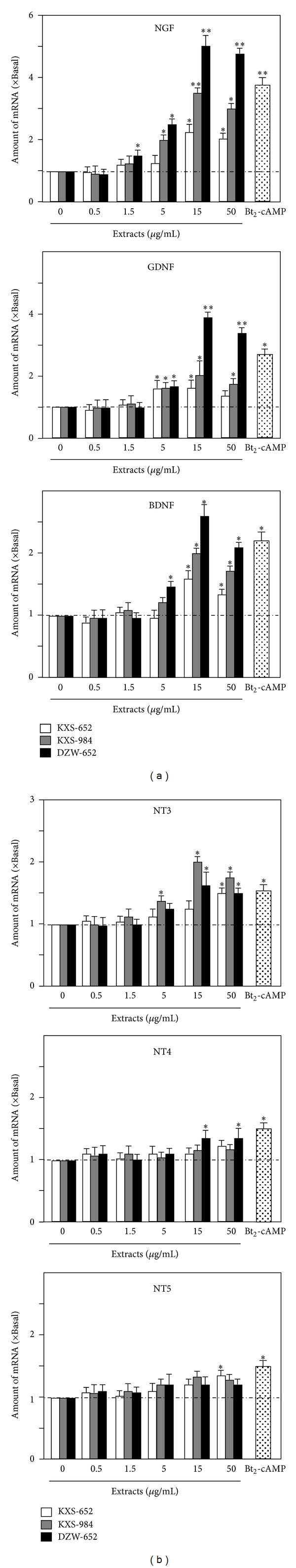
KXS stimulates the mRNA expression of neurotrophic factors in astrocytes. The mRNA expression levels of neurotrophic factors (NGF, GDNF, BDNF, NT3, NT4, and NT5) in astrocytes were analyzed. The astrocytes were treated with different formulations of KXS (0.5–50 *μ*g/mL) for 24 hours. The mRNA was determined by quantitative PCR. Primers for neurotrophic factors were listed in Supplementary Table 1. Available online at http://dx.doi.org/10.1155/2013/731385 Bt_2_-cAMP (1 mM) was used as a control. Data are expressed as *x* Basal where the value of control is set as 1 and in Mean ± SEM, *n* = 4.

The effect of the three formulations of KXS on the expression of neurotrophic factors was evaluated by quantitative PCR, which included NGF, BDNF, GDNF, NT3, NT4, and NT5. The specific primers of these neurotrophic factors were listed in Supplementary Table 1. Astrocytes cultured at DIV 12 were treated with KXS from 0.5–50 *μ*g/mL for 24 hours, and then the amounts of mRNA were analyzed. The KXS treatment significantly increased the mRNA levels of NGF, BDNF, GDNF, and NT3 in dose-dependent manners (Figure 2). For NGF, the mRNA expression level showed over 2-fold enhancement under the treatment of KXS from 5 to 50 *μ*g/mL, while the DZW-652 showed the best effect (~4-fold) at concentration of 15 *μ*g/mL. For BDNF, DZW-652 increased the mRNA expression level over 2.5-fold. The best effect of DZW-652 could be also observed in GDNF induction. For NT3, K-984 showed better effect (~2-fold). For NT4 and NT5, there were slight changes observed after KXS treatment (Figure 2). Thus, KXS having a high ratio of Ginseng Radix et Rhizoma and Polygalae Radix could benefit the expression of neurotrophic factors, as this was in DZW-652 formulation.

**Figure 3:**
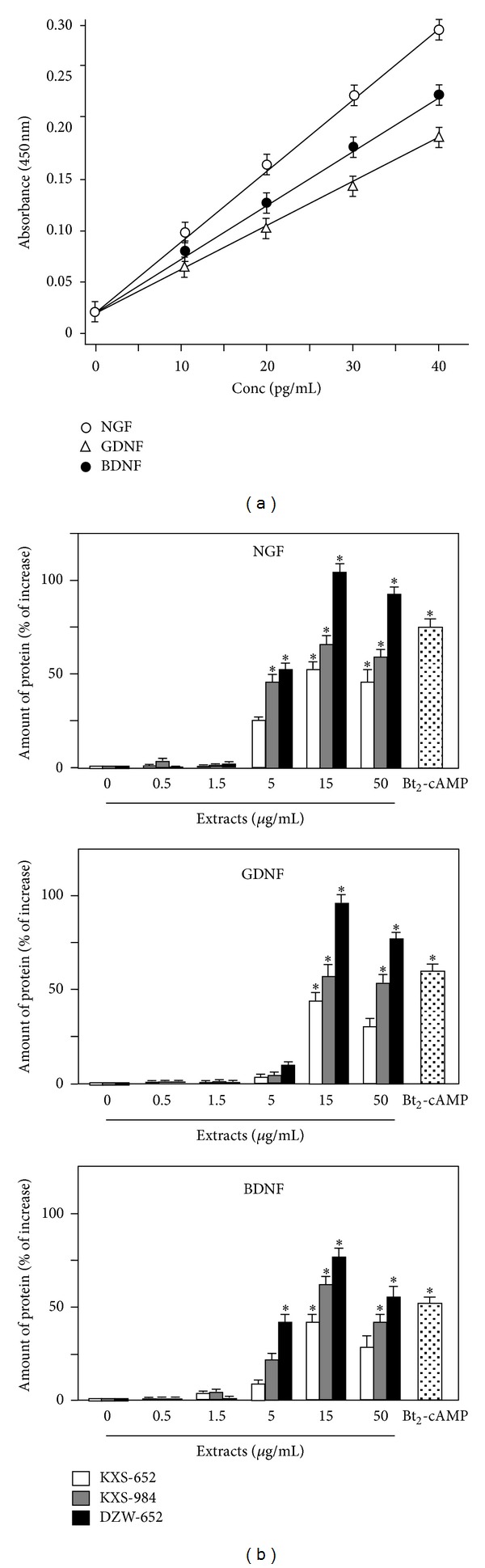
KXS stimulates the secretion of neurotrophic factor in astrocytes. (a) Different concentrations of neurotrophic factors (0–40 pg/mL) were applied onto ELISA kit, and the absorbance was detected at wavelength of 450 nm. The calibration curve of each target protein was constructed by plotting absorbance versus the concentration of each target protein. Each calibration curve was derived from 5 data points. (b) Astrocytes were treated with KXS (0.5–50 *μ*g/mL) and Bt_2_-cAMP (1 mM) for 48 hours. Then, the media were collected for analysis of NGF, BDNF, and GDNF with ELISA kit. Data are expressed as percentage of increase to control (no drug treatment, that is, 0 extract here). Values are showed as the Mean ± SEM, *n* = 3.

**Figure 4:**
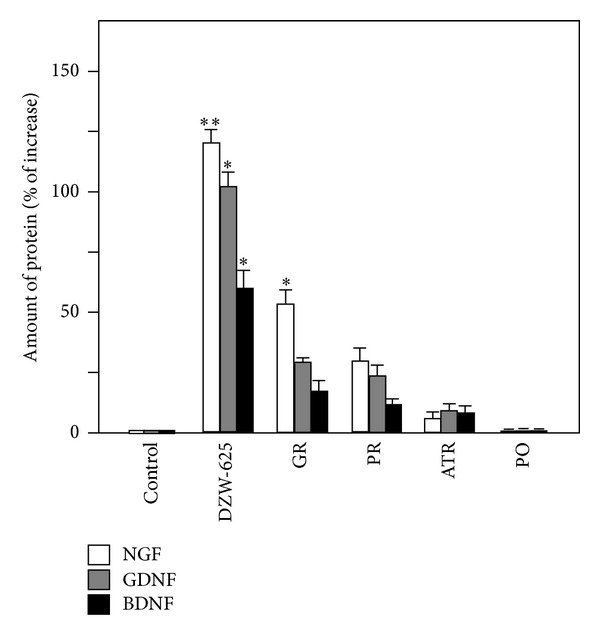
The comparison of KXS and single herb in stimulating the secretion of neurotrophic factors in astrocytes. Astrocytes were treated with DZW-652 (15 *μ*g/mL), the extract of Ginseng Radix et Rhizoma (4.5 *μ*g/mL), the extract of Polygalae Radix (3 *μ*g/mL), the extract of Acori Tatarinowii Rhizoma (3 *μ*g/mL), and the extract of Poria (4.5 *μ*g/mL) for 48 hours, and then the media were collected to determine the amount of NGF, BDNF, and GDNF. Data are expressed as percentage of increase to control (no drug treatment, that is, 0 extract here). Values are showed as the Mean ± SEM, *n* = 3. Abbreviations: GR: Ginseng Radix et Rhizoma; PR: Polygalae Radix; ATR: Acori Tatarinowii Rhizoma; PO: Poria.

The KXS-induced gene expression was robust for NGF, BDNF, and GDNF, and thus, the proteins of these factors in astrocytes' conditioned medium were further determined. Firstly, standard curves of three target proteins in ELISA assays were made by different concentrations of protein standards. NGF, BDND, and GDNF exerted good linearity in a range of 0~40 pg/mL (Figure 3(a)), and the square of linear correlation coefficient was all over 0.95. The recovery of spiked protein was greater than 90%. The coefficient of intra and interassay was over 0.90. In astrocytes treated with KXS extracts, the induction of NGF, GDNF, and BDNF was determined by ELISA. The amount of NGF of cultured astrocytes was 4.92 pg/mg proteins, while the value of BDNF and GDNF was 2.39 pg/mg protein and 21.6 pg/mg proteins, respectively. Under the treatment of KXS, the expressions of NGF, GDNF, and BDNF were increased in dose-dependent manners (Figure 3(b)). At 15 *μ*g/mL of KXS in all cases, the inductions were significant higher than that of the control. The treatment of DZW-652 exerted the best stimulation in neurotrophic factor secretion: a maximal induction at 100% increase compared to control was revealed under 15 *μ*g/mL of DZW-652 (Figure 3(b)). In addition, the inducing effect of KXS-984 was slightly better than that of KXS-652 (Figure 3(b)). Here, the application of Bt_2_-cAMP was used as a control. In addition, the water extracts of four individual herbs were tested for their role in the induction of neurotrophic factors, and the effects were not significant. Here, the water extracts of Ginseng Radix et Rhizoma and Polygalae Radix could slightly stimulate the expression of NGF and GDNF (Figure 4), while the other inductions were very small. In contrast, a combined herbal mixture (i.e., DZW-652) showed robust induction.

### 3.2. The Signaling of KXS-Induced Neurotrophic Factor Expression

**Figure 5:**
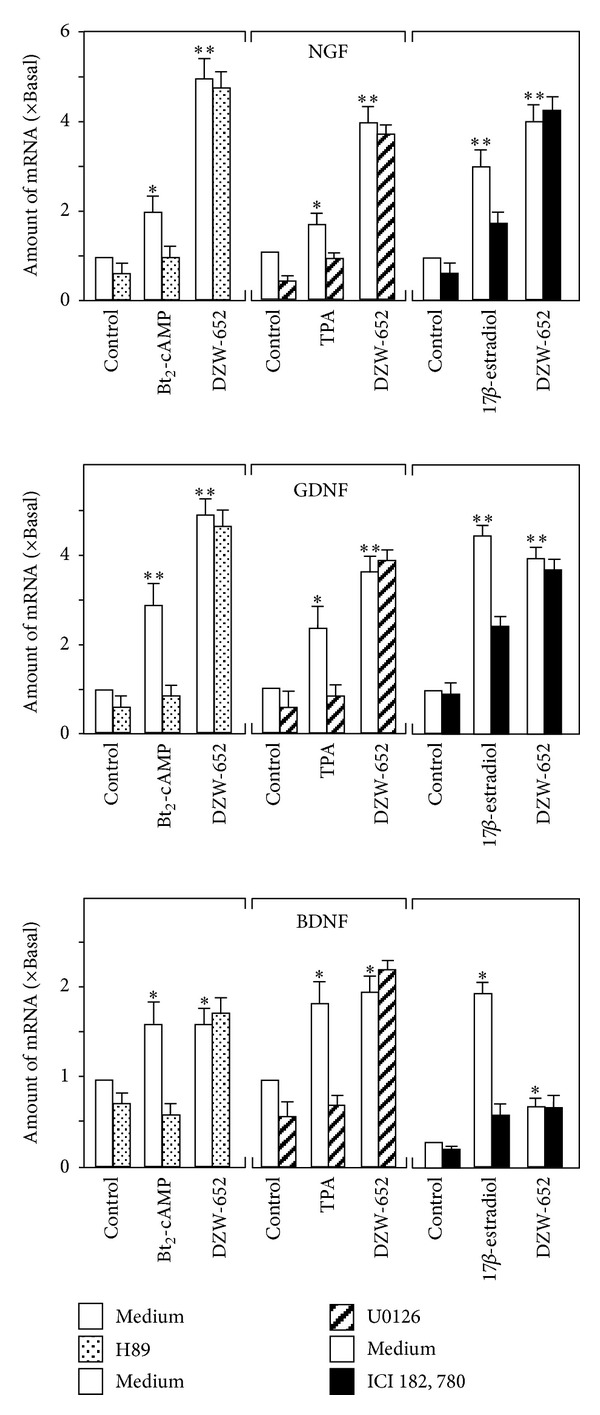
Effect of cAMP, MAPK, and estrogen signaling pathway on the expression of neurotrophic factors. Astrocytes were pre-treated with H89 (2 *μ*M; the PKA inhibitor), U0126 (10 *μ*M; the MEK1/2 and PKC inhibitor), and ICI 182, 780 (1 *μ*m; the estrogen receptor inhibitor) for 3 hours and then treated with Bt_2_-cAMP (1 mM), TPA (100 nM), 17*β*-estradiol (10 nM), and DZW-652 (15 *μ*g/mL) for 24 hours. The mRNA expression levels of neurotrophic factors in astrocytes were analyzed. Data are expressed as *x* Basal where the value of control is set as 1 and in mean ± SEM, where *n* = 4.

To search for the possible mechanism of KXS-induced neurotrophic factor expression on astrocytes, the known signaling pathways, including cAMP-dependent, MAPK-dependent, and estrogen receptor-dependent [14–17], were probed here. The cultured astrocytes were treated with the corresponding agonists, for example, Bt_2_-cAMP activating cAMP-dependent signal TPA activating MAPK-dependent signal, and 17-*β*-estradiol activating estrogen receptor-dependent signal. The application of these positive inducers activated robustly the mRNA expressions of NGF, GDNF, and BDNF (Figure 5) in cultured astrocytes. As expected, the application of H89, U0126, and ICI 182, 780 significantly reduced the inductions of Bt_2_-cAMP, TPA, and 17-*β*-estradiol, respectively. However, the DZW-652-induced gene expressions were not affected by these signaling inhibitors (Figure 5). Thus, the role of KXS in inducing the expressions of neurotrophic factors was novel, at least not belonging to any known signaling pathways. 

## 4. Discussions

Astrocyte is the prevailing glial cell in the central nervous system and constitutes the blood brain barrier, which is an essential component in protecting the inner environment of the brain. More importantly, there is an intensive bidirectional communication between neurons and glial cells at the synapses, which leads to a concept of “tripartite synapse,” that is, a presynaptic neuron, a postsynaptic neuron and glial cells [18]. Astrocyte plays a role in the uptake of neurotransmitter from synaptic cleft, the synthesis of neurotransmitter precursors, and disposal of excess neurotransmitter [19]. On the other hand, astrocyte is an important regulator for the modulation of neurotrophic factors [20, 21]. 

Neurotrophic factors are a family of proteins responsible for the growth and survival of neurons during development and for maintenance of adult neurons, for example, NGF, BDNF, GDNF, NT3, and NT-4/5. The deficiency of neurotrophic factors in brain has been regarded as a biomarker for depression. The chronic stress in animals induced decreased synthesis of neurotrophic factors, which subsequently caused atrophy of neurons in hippocampus [22, 23]. In parallel, the stress was also reported to decrease the expression of BDNF in CA3 pyramidal and dentate gyrus of hippocampus in rat [24]. These observations led to the hypothesis that downregulation of BDNF contributed to an accelerated atrophy of CA3 neurons. Here, the treatment of KXS not only enhanced the expression of neurotrophic factors, for example, NGF, BDNF, GDNF, and NT3, but also stimulated the secretion of these neurotrophic factors in cultured astrocytes, which was in line to antidepression function of KXS in rats [12]. In depressive rats, total saponins of Ginseng Radix et Rhizoma reversed the stress-induced decreased BDNF level, and in parallel ginsenoside, Rg_1_ up-regulated the BDNF signaling pathway in hippocampus of mice [25, 26]. In addition, 3, 6′-disinapoyl sucrose derived from Polygalae Radix reversed the reduced BDNF levels in stress-induced depressive rats [27], while saponin from Polygalae Radix enhanced the production of NGF in rat astrocytes [28]. Eugenol derived from Acori Tatarinowii Rhizoma increased BDNF mRNA expression level in hippocampus of mice [29]. Among the tested three KXS formulations, the amount of Ginseng Radix et Rhizoma and Polygalae Radix was the highest in DZW-652, which therefore might explain the best effect of this ratio in enhancing neurotrophic factor expression. 

There are several possibilities of having three formulations of KXS in history. Due to a lack of printing technologies in Tang Dynasty, the original edition of *Beiji Qianjin Yaofang* (652 A.D.) had never been seen. The earliest edition of *Beiji Qianjin Yaofang*, which could be found today, was edited by Lin Yi and Gao Bao-heng in Song Dynasty, who described one of the KXS formulae, KXS-652 with a ratio of 1 : 1 : 25 : 50 (Ginseng Radix et Rhizoma : Polygalae Radix : Acori Tatarinowii Rhizoma : Poria). Meanwhile, a herbal formula named Ding-Zhi-Wan (DZW-652) was also described having the herbal ratio of 3 : 2 : 2 : 3 in the same book. In addition, the composition of another herbal formula, named KXS-984, was recorded in *Yi Xin Fang* written by Nima Yasunori from Japan in 984 A.D. (Song Dynasty). The author recorded the herbal ratio of 1 : 1 : 1 : 2 for KXS-984 and cited *Beiji Qianjin Yaofang*. Despite the variations of herbal composition, all three KXS formulae have been used to treat the diseases with symptoms of depressed mood and morbid forgetfulness [30, 31]. The disappearance of the original manuscript of *Beiji Qianjin Yaofang* in Tang Dynasty might also be a reason for emerging different KXS formulae. More realistically, the original KXS formula might have been rearranged by other TCM practitioners according to syndrome differentiation. 

The mechanism of KXS in stimulating mRNA expression levels of neurotrophic factors was unknown. It has been reported that cAMP-dependent pathway, MAPK-dependent pathways, and estrogen signaling pathway might be included in neurotrophic factor expression [14, 15]. Here, the effect of KXS in stimulating neurotrophic expression could not be inhibited by the corresponding inhibitors, that is, H89, U0126, or ICI 182, 780. KXS is a herbal extract having multichemicals, which therefore could have different roles in stimulating neurotrophic factor expression via multiple signal transduction pathways or unidentified kinase(s), that is, not only the reported cAMP-, MAPK- and estrogen-dependent pathway. Thus, we hypothesized that KXS might exert functions in stimulating neurotrophic factor expression through multitargets and multisignaling pathways. Some new pathways might be also involved instead of the single reported well-known pathways. Other method, such as phosphorylation proteomics, might be applied for further studies in the future.

## 5. Conclusions

The treatment of KXS in cultured astrocytes increased both the synthesis and the release of neurotrophic factors, including NGF, BDNF, and GDNF. Although the action mechanism was not fully revealed, this result was in parallel to our previous report that KXS was effective in treating antidepression. In line to the historical usage, this chemically standardized herbal extract could serve as alternative medicine or health food supplement for patients suffering from depression.

## Supplementary Material

Supplementary Figure 1: Chemical fingerprint chromatograms of KXS formulae. (A): Fingerprint chromatograms of KXS formulae were made by HPLC-DAD at wavelength of 330 nm. The identification of 3, 6'-disinapoyl sucrose (1), **α**-asarone (8) and **β**-asarone (7) were shown in the chromatogram. (B): Fingerprint chromatograms of KXS were made by HPLC-MS/MS method at negative scan mode. The identification of ginsenoside Rg1 (2), Re (3), Rb1 (4), Rd (6) and pachymic acid (9) were shown in the chromatogram. The internal marker control was astragaloside IV (5). The experimental details were fully described in *[*13*]*. Representative chromatograms are shown, *n* = 3.

## Figures and Tables

**Table 1:** Historical record of different KXS formulae.

Notation^a^	Record	Ratio			
		GR	PR	ATR	PO
KXS-652	*Beiji Qianjin Yaofang* ^ b^	1	1	25	50
KXS-984	*Yixin Fang* ^ c^	1	1	1	2
DZW-652	*Beiji Qianjin Yaofang* ^ b^	3	2	2	3

^a^The notation of KXS was corresponding to the years of recording.

^b^ *Beiji Qianjin Yaofang* was written by Sun Simiao in 652 A.D, which was re-edited in 1066 A.D.

^c^ *Yixin Fang* was written by Nima Yasunori in 984 A.D.

Abbreviations: GR: Ginseng Radix et Rhizoma; PR: Polygalae Radix; ATR: Acori Tatarinowii Rhizoma; PO: Poria.
