# Supplementary material for: Kai-Xin-San, a Chinese Herbal Decoction Containing Ginseng Radix et Rhizoma, Polygalae Radix, Acori Tatarinowii Rhizoma, and Poria, Stimulates the Expression and Secretion of Neurotrophic Factors in Cultured Astrocytes
Source: Evid Based Complement Alternat Med. 2013 Oct 3;2013:731385. doi: 10.1155/2013/731385 (PMC3814066; doi:10.1155/2013/731385)
Supplement: Supplementary file 1 — Supplementary Figure 1: Chemical fingerprint chromatograms of KXS formulae. (A): Fingerprint chromatograms of KXS formulae were made by HPLC-DAD at wavelength of 330 nm. The identification of 3, 6'-disinapoyl sucrose (1), α-asarone (8) and β-asarone (7) were shown in the chromatogram. (B): Fingerprint chromatograms of KXS were made by HPLC-MS/MS method at negative scan mode. The identification of ginsenoside Rg1 (2), Re (3), Rb1 (4), Rd (6) and pachymic acid (9) were shown in the chromatogram. The internal marker control was astragaloside IV (5). The experimental details were fully described in [13]. Representative chromatograms are shown, n = 3. [file 731385.f1.ppt]

## Slide 1
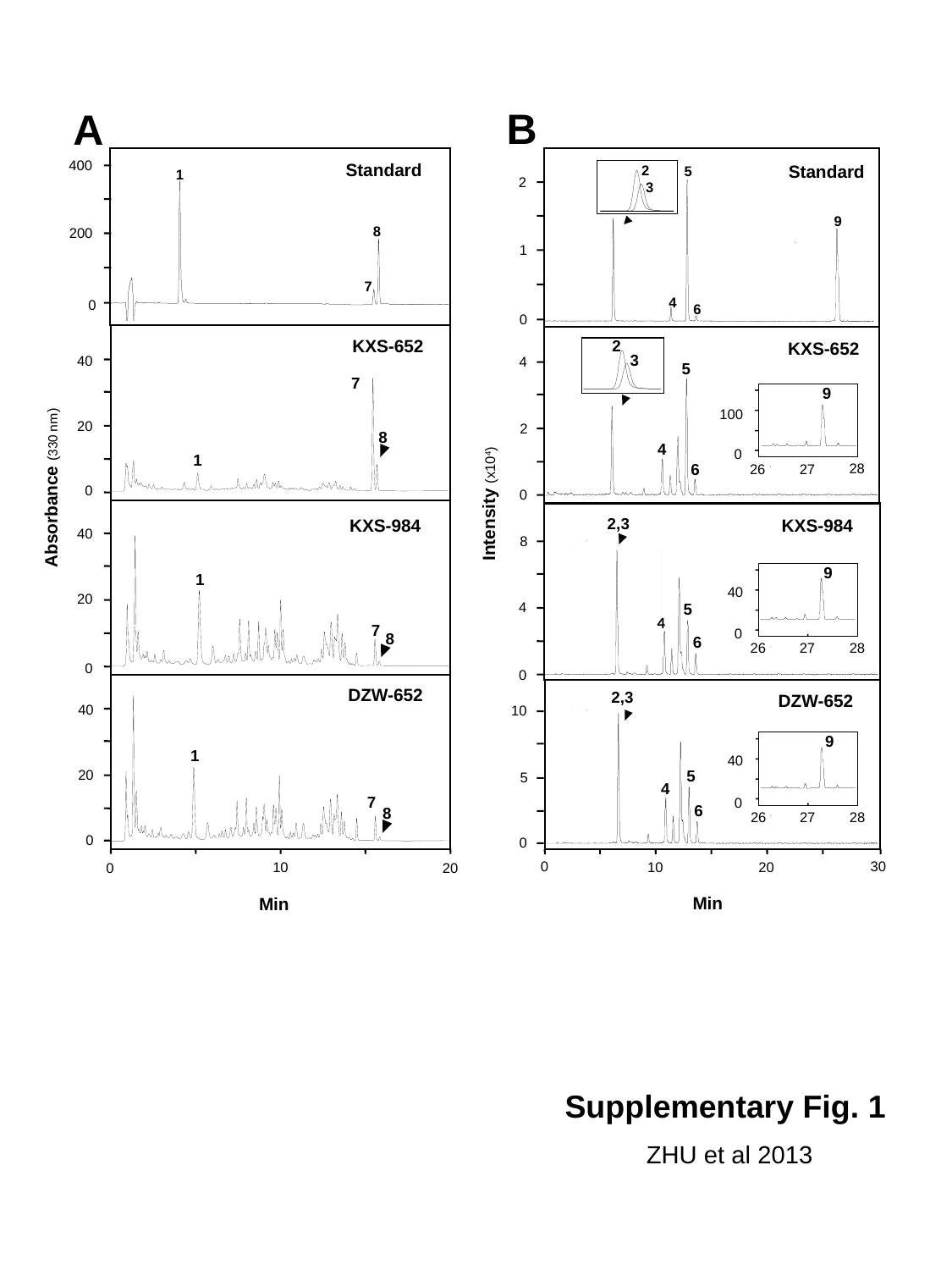

B
A
400
Standard
Standard
 1
2
5
2
3
9
8
200
1
7
4
0
6
0
KXS-652
2
KXS-652
3
40
4
5
7
9
100
Absorbance (330 nm)
20
2
8
4
0
 1
28
6
26
27
0
0
Intensity (x104)
2,3
KXS-984
KXS-984
40
8
9
 1
 40
20
4
5
4
7
0
8
6
28
26
27
0
0
DZW-652
2,3
DZW-652
40
10
9
 1
 40
5
20
5
4
7
0
6
8
28
26
27
0
0
0
30
10
10
20
20
0
Min
Min
Supplementary Fig. 1
ZHU et al 2013
